# Advocating for incorporating public veterinary health care into human health systems

**DOI:** 10.3389/fvets.2025.1600839

**Published:** 2025-10-20

**Authors:** Laurie Meythaler-Mullins, Cheryl Eia, Allyce H. Lobdell, Danielle M. Frey

**Affiliations:** ^1^College of Veterinary Medicine and Biomedical Sciences, Colorado State University, Fort Collins, CO, United States; ^2^College of Veterinary Medicine, Center for Food Security and Public Health, Iowa State University, Ames, IA, United States

**Keywords:** public health veterinary care, rabies, Alaska, dogs, canines

## Abstract

In the first study to examine veterinary services in rural Alaska, this paper focuses on the use of a public health veterinary program embedded in the human health care system as a means of improving human health, specifically in rural villages such as the Yukon-Kuskokwim Delta of Alaska (YK Delta). The YK Delta is home to approximately 23,000 people living in 58 distinct rural communities, each of which is a federally recognized Tribe. Dogs play a particularly important role in these tribal communities. However, access to veterinary services is extremely limited in the region. Bringing together data from the Hub Outpost Project (HOP) which was designed to deliver preventative, public health veterinary services throughout the region, a meta-synthesis on relevant topics in similarly situated regions, and cost data, this paper examines three critical veterinary public health issues affecting the YK Region: (1) human exposures to rabies, (2) dog bite injuries and dog overpopulation, and (3) how a veterinary preventative medicine program like HOP can provide a cost-effective way to improve human health and reduce the cost to individuals and the community from these risks. The HOP model demonstrates effective and consistent veterinary preventative care in that it increased the number of vaccinated dogs, thereby reducing rabies exposure risk to humans. As demonstrated in this paper, the authors believe the education and population control provided by programs like HOP can decrease the number of dog bites and dog overpopulation. The authors strongly advocate for the implementation of this One Health model of veterinary service within the established human health system to maintain consistency and effectiveness.

## 1 Introduction

Sufficient access to veterinary medicine is a critical component of public health. Occupying the interface between animal and human health, veterinary medicine’s focus is two-fold; first, providing care to improve the health and welfare of animal patients and second, protecting public health by reducing the human health risks posed by contacts between humans and animals. When access to veterinary medicine is not readily available, adverse health consequences related to human-animal contacts may increase, often with far-reaching impacts affecting the physical, emotional, and financial well-being of individuals, families, communities, organizations and society.

In the Yukon-Kuskokwim Delta of Alaska (YK Delta), dogs have played an important role in Alaska Native culture for centuries. Not only do dogs provide companionship, but they are also woven into the social fabric of everyday life. However, due to geographic, transportation, and socioeconomic barriers, there is often no veterinary care available to residents in this region. Because of this, residents are exposed to various animal-related health threats including zoonotic pathogens.

As the first study to examine veterinary services in rural Alaska, the authors of this paper use examples from a program operated in the Yukon-Kuskokwim Delta of Alaska (YK Delta), the Hub Outpost Project (HOP). Using a One Health Framework, HOP demonstrates how preventative veterinary medicine can mitigate impacts on individual human and community health and wellness. The authors focus on human exposure to rabies virus from dogs, regionally elevated rates of dog bite injuries, and dog overpopulation.

HOP is a preventative veterinary medicine model developed in 2017. The authors will discuss what was learned from implementing this model by demonstrating how the financial cost of operating a preventative veterinary medicine program such as HOP may be more cost-effective than the current approach undertaken by regional human health care providers. Further, the authors describe how this model also reduces the costs incurred by individuals and societies in these underserved regions. Through examining these costs, the authors strongly recommend incorporating public health veterinarians into human healthcare systems.

### 1.1 Background

Alaska and YK Delta region, distinct from the rest of the United States, also has a distinct culture. It is important to understand the nuances of life and culture in the state and region to understand the distinct threats to human and animal health experienced there.

#### 1.1.1 Alaska

**Figure 1:**
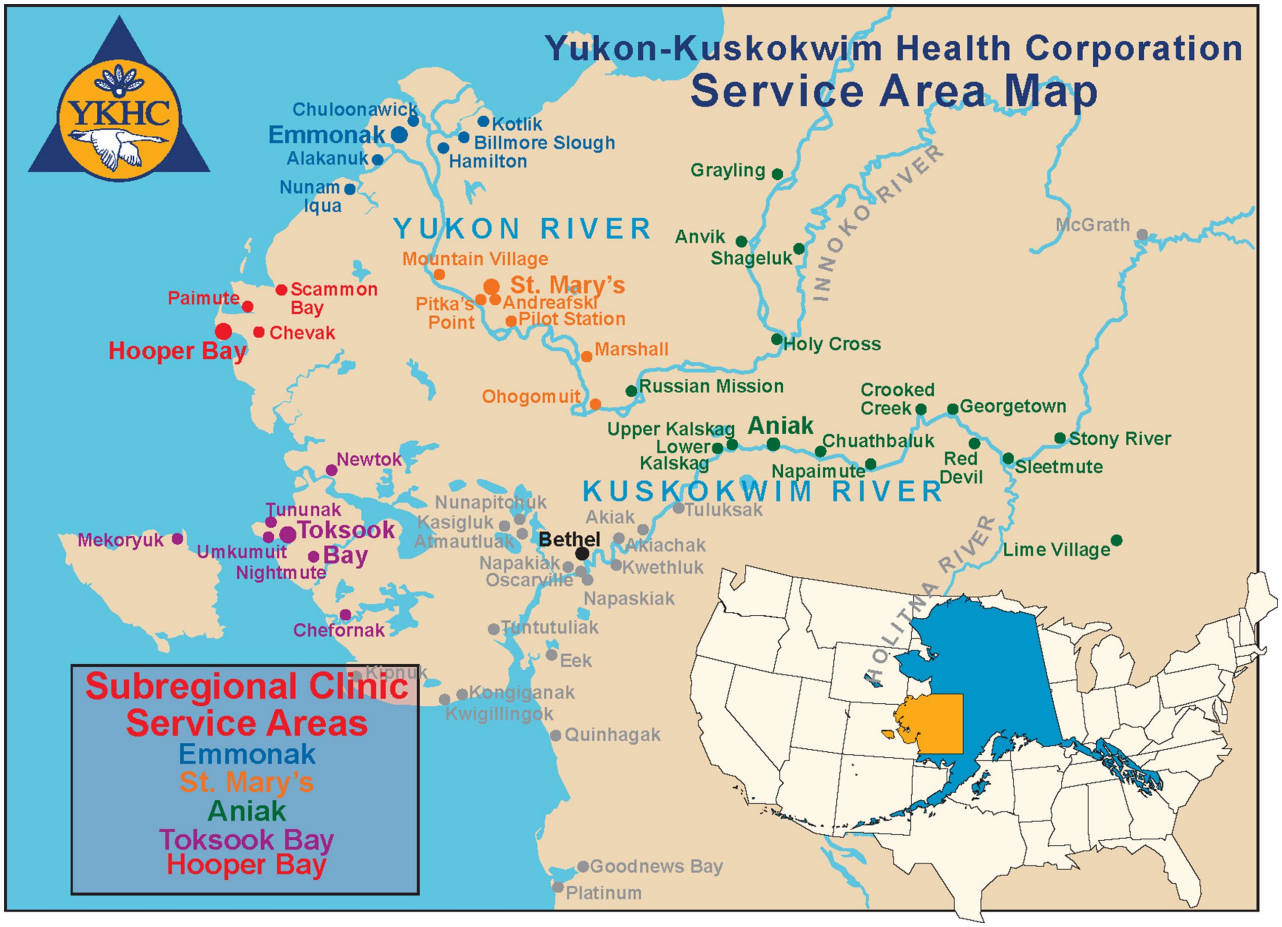
Yukon Kuskokwim health corporation service map.

The state of Alaska is geographically separate and distinct from the rest of the United States, which is often referred to as the “lower-48” by Alaskans. Located in the northwest corner of the North American continent, Alaska is the largest state in the United States, covering a land area one-fifth the size of all the lower-48 states as shown in Figure 1 (1). Alaska is vast, with 14 mountain ranges and 17 of the 20 highest peaks in the United States, as well as tundra, forests and a long coastline with islands scattered in the sea. With 3,000 rivers and over 3 million lakes, fresh water is abundant. The sheer size of the state, expansive wilderness areas, and many natural barriers make air travel the primary means of transportation throughout much of the state (2). This is especially true for travel into and out of Alaska Native villages (2), most of which are not connected to the state’s limited road system. It is notable that, despite its size, Alaska has only 17,637 miles of public roads. As a comparison, Texas, the second largest U.S. state by area, has 323,364 miles of public roads, and Rhode Island, the smallest state by area, has 6,571 miles of public roads (3).

Factors such as location, climate, geography, and population create living conditions in rural areas of Alaska that more closely resemble those of rural areas in the Northwest Territories of Canada than anywhere else in the United States (4). Alaska is directly west of the Northwest Territories of Canada, and they share a similar climate, terrain and limited access by roads. This is the first study examining veterinary services in rural Alaska. Several papers describe veterinary care in the Northwest Territories, and these are used as comparisons to the findings in this paper.

#### 1.1.2 The YK Delta region

The YK Delta region occupies the southwest portion of Alaska as shown in Figure 1. It is comprised of tundra, mountains, and wetlands. Two major rivers, the Yukon and the Kuskokwim, flow through the region on their way to the Bering Sea. This region, with an area of 75,000 square miles, is approximately the same area as the state of Oregon. The region has a population of approximately 23,000 people who live in 58 distinct rural communities, each home to a distinct tribal nation, each of which is a federally recognized tribe. These communities may vary in population from just 25 to 1,000 people. The largest community, Bethel, is the exception with 7,000 people (5).

Bethel is the regional service and transportation center of the YK Delta. The Bethel Airport provides flight services to Alaska’s largest city, Anchorage and to villages throughout the region. None of the villages are accessible by road, that is, there are no roads from the villages to Bethel. From the villages, Bethel is only accessible by airplane, boat (in the warmer months), snowmobile (in the winter months), and four-wheeler. Food and supplies are delivered to the villages by air freight, yet these are limited and expensive. Therefore, subsistence methods, the reliance on hunting, fishing, harvesting, and processing of terrestrial and marine mammals, fish, plants, and other natural resources (6, 7) to meet essential and cultural needs, are important in the traditional culture and still remain crucial to the everyday life of Alaska Native Tribes.

This region is home to three cultures: Yup’ik, Cup’ik, and Athabascan. Traditionally, the Yup’ik have lived throughout the region; the Cup’ik have called Nunivak Island and coastal villages home; and the Athabascans have traditionally resided along the Yukon River and interior villages. The Yup’ik language is the first language of more than 14,000 residents, making it the most widely spoken Alaska Native language today (5).

Dogs occupy a special role in Alaska Native culture generally and have for centuries, serving not only as companions but also in assisting with transportation, protection and hunting. In the YK Delta specifically, dog teams were still used for hunting, trapping and transportation through the 1950’s. As snowmobiles took over for transportation, dogs were still kept in communities to teach Native ways of knowing. They have been used to connect indigenous youth to their culture, in suicide prevention and substance abuse programs (8). There is a vibrant dog mushing community in the YK Delta with multiple established races, including the Kuskokwim 300, the world’s premier middle distance sled dog race (9).

The YK Delta’s unique characteristics set it apart from the rest of Alaska and this, along with its primarily rural make up, have distinctively impacted the general life and health in the region, access to health care, and public health. The role of dogs in these communities further impacts each of these issues.

#### 1.1.3 Healthcare in the YK Delta

Approximately 82% of the population in the region are *Alaska Native* (5), whose healthcare is provided through Indian Health Services (IHS), an agency within the U.S. Department of Health and Human Services, along with the Yukon-Kuskokwim Health Corporation (YKHC), a Tribal Organization. Headquartered in Bethel, YKHC is authorized by the tribal governing councils to provide health services on behalf of Indian Health Services and administers a comprehensive healthcare delivery system for all 58 communities in the YK Delta. Forty-one village clinics staffed by Community Health Practitioners provide care in the villages. Five Sub-Regional Clinics are available to take referrals from village clinics and provide a higher level of care. The YK Delta Regional Hospital, located in Bethel, provides inpatient and outpatient care and treatment in a variety of specialties. It offers several other health programs, including substance abuse, and additional facilities for things such as mental health treatment and housing for patients traveling to Bethel for medical care (10).

#### 1.1.4 Challenges to providing veterinary medicine in rural Alaska

Providing veterinary care in remote, sparsely populated areas poses challenges not only in large areas of Alaska, but also on tribal lands both in the lower-48 states and throughout the Arctic. The United States Department of Agriculture has recognized that there is a shortage of veterinarians in rural areas across the United States and has taken measures to work with states to identify shortage areas and provide incentives to veterinarians to practice in those areas through the Veterinary Medicine Loan Repayment Program (VMLRP) (11). This program, with a focus on food animal veterinary medicine and supporting disciplines, does not adequately address the need for veterinary medicine in those rural communities in which residents rely on small scale subsistence for food and where dogs and other companion animals have an important role in the community and culture. Additionally, the VMLRP does not allow focus on companion animal care to address areas and concerns where residents are exposed to various (companion) animal-related health threats including zoonotic pathogens.

Most rural Alaska communities are too small to support a local veterinary practitioner on their own without some form of outside support. The costs associated with travel to the communities make it economically unsustainable for veterinary practices to provide regular service to the communities. This results in access to veterinary services being limited to those who can afford to fly their animal to a larger city, often at a cost ranging from hundreds to thousands of dollars, a prospect unaffordable to most dog owners (4). Because of this, most animals in the YK Delta villages do not receive preventive veterinary treatments and procedures that are critical to protecting humans.

This can directly result in several unwelcome outcomes such the risk of rabies exposure in village residents through contact with unvaccinated dogs which may have encountered rabid wildlife (12). There is also the risk of unplanned litters of puppies due to lack of access to spay/neuter care leading to an overpopulation of stray and abandoned dogs in villages and increasing the risk of human exposure to disease, parasites, and dog bites (13).

In addition to providing healthcare services, veterinarians also have the knowledge and skillsets to mitigate public health threats by providing education and knowledge on topics including food safety. This is becoming increasingly important of the effects of climate change on traditional methods of food preservation and storage and rural Alaskans’ heavy reliance on a subsistence lifestyle. The hunting and gathering activities practiced by many rural residents may place them at a greater risk of acquiring zoonotic infections than their urban counterparts. Many zoonotic diseases are known to occur in wild animals caught for food in Alaska including brucellosis, toxoplasmosis, trichinellosis, giardiasis/cryptosporidiosis, echinococcosis, rabies and tularemia (14).

Alaska has two state public health veterinarians whose duties include disease surveillance, coordinating emergency response in the event of a disease outbreak or natural disaster. They coordinate with state and local partners to ensure animal welfare standards are met and work with food producers to meet requirements for national animal health certification programs (15). Yet the size and population distribution in the state limits the efficacy and impact of a staff of this size. It does not have the capacity to provide on-the-ground veterinary services in all its rural areas.

#### 1.1.5 The Hub Outpost Project (HOP)

The Hub Outpost Project (HOP) was envisioned in 2017 and in 2018 a regional partnership between HOP and the YKHC Office of Environmental Health and Engineering (OEHE) was developed to introduce a system to address the lack of veterinary care in this region. While the human healthcare system in this rural region is well-established, no such targeted system existed to address the lack of veterinary care. The HOP model was designed with input from YKHC and based on its human healthcare delivery model. Using a hub-and-spoke design model, the project deploys a veterinary team from the “hub” of Bethel to strategically identified “spoke” communities. Veterinary field clinics are established in these outlying spoke communities. Each spoke community, in turn, is accessible to an additional five to ten communities. HOP began delivering preventative, public health veterinary services to the YK Delta in 2019.

#### 1.1.6 Dog population in the YK Delta

Historical evidence demonstrates that dogs have been a part of Alaska Native life for thousands of years (16). As is true in many cultures, dogs provide companionship but also are woven into the social fabric of everyday life in these Alaska Native communities. In the villages served by the HOP, people still choose to live with dogs, even though dogs have lost some of their traditional working roles.

Generally, dogs in YK Delta can be divided into two main groups: “owned” and “unowned.” The owned dog population is dependent upon humans for food, water, and shelter and includes dogs who are kept in a limited area (e.g., in a dog yard), and those that are free to roam without human supervision, or “loose,” as the locals refer to them. A single dog may have one owner or may be a “community dog,” having more than one owner. Unowned dogs (often referred to as “stray”) do not have an owner but may still depend upon humans directly or indirectly for food, water, and shelter.

Free-roaming or “loose” dogs, both owned and unowned, are commonly referred to as the free-roaming dog population (17). Understanding the loose dog population is particularly important to understanding public health issues related to rabies exposure, dog bites and overpopulation, as the lack of physical restriction allows these dogs to mate and reproduce, to encounter wildlife, and have variable socialization.

#### 1.1.7 Rabies

The rabies virus causes one of the world’s deadliest diseases, with a fatality rate of nearly 100% if not promptly treated (18). Rabies is endemic in most countries, including the US, where wildlife species (i.e., skunk, fox, raccoon, mongoose, and arctic fox) are the main reservoirs (19). Worldwide exposure to rabies virus disproportionately affects people in poor, rural areas, especially children (20, 21).

Rabies is transmitted from animals to humans through direct contact with the saliva, brain, or nervous system tissue from an infected animal. The virus enters the body through broken skin or the mucous membranes and makes its way to the central nervous system. In animals, rabies can only be diagnosed after death by detecting the virus in the brain. Once clinical symptoms appear, rabies is always fatal. Worldwide, 99% of all human cases are the result of being bitten by a domestic dog (18).

Vaccinating dogs and other animals against rabies is a common rabies prevention strategy. The World Health Organization (WHO) notes that mass dog vaccination programs are the most cost-effective strategy for preventing dog rabies in people because it stops the transmission of rabies at its source (18). In the U.S., rabies vaccines are licensed for use in several domestic species including dogs, cats, ferrets, horses, and sheep. Other livestock and zoo animals may also receive rabies vaccines (22).

#### 1.1.8 Dog bites

According to the American Veterinary Medical Association (AVMA), in 2023, there were between 84–89 million dogs in the United States and 45% of homes had at least one dog. The AVMA report further informs that dogs bite over 4.5 million people each year, with children being the most bitten population. This issue also comes with a cost—in 2023, insurers paid $1.2 billion in claims related to dog bite injuries, with the average cost of a dog bite claim at $58,545 (23).

#### 1.1.9 Population control

Where free-roaming dogs exist in high densities, they can be considered an issue in terms of public health (e.g., transmission of rabies and other zoonotic pathogens), impacts to the wildlife and the environment, and the animals’ own welfare state (24–26).

## 2 Materials and methods

To conduct this study, a multiple methods methodology was used. First, information gathered for and from HOP operations was identified and incorporated. Second, a meta-synthesis was conducted with literature on relevant topics in similarly situated regions. Third, cost data and information were gathered from governmental websites, HOP operations data, and authors’ personal experiences. Finally, these sources of information were assessed all together to solidify a larger picture of the both the needs and potential solutions to the overlapping issues at hand.

### 2.1 HOP experiential data

During the HOP’s time operating, team members in the field gathered information from a variety of sources to assist in measuring the project’s impact. This includes narratives and survey data. Survey data comes from K-12 school visits by HOP in the YK Delta communities and formal censuses of dogs in three villages in the YK Delta.

To help provide an approximation of dog populations in the region, three censuses were conducted by HOP. The first two censuses were completed following confirmed cases of animal rabies in the villages of Quinhagak and Napaskiak. The third census was completed during a scheduled HOP visit to Akiak.

As a task within its program operations, HOP also collected information from YKHC OEHE on rabies exposures. This information informed where HOP would be most impactful to visit and hold clinics. The YKHC also provided information and data on rabies investigations and post-exposure prophylaxis.

Census and survey data was analyzed in the statistical software R and Excel. Descriptive statistics were conducted in R while the short qualitative data from open text questions was analyzed thematically using Excel. YKHC data was not further analyzed but used as was presented by the organization.

### 2.2 Meta-synthesis

The authors systematically researched, analyzed, and synthesized existing academic literature related to human exposures to rabies, injuries due to dog bites, dog overpopulation dynamics, and access to veterinary care, including similarly situated geographic regions. Specifically, from October to December of 2024, authors searched PubMed, Google Scholar, and governmental websites for information and papers on these topics. For information on rabies topics, PubMed was the primary database used. Search terms used included “rabies” and “cost,” “exposure,” “prevention,” “control,” “post exposure prophylaxis” or “PEP” and “exposures United States.” The authors reviewed a number of papers focused on various countries as well as regions of the U.S. where the cost of rabies control had been estimated for a particular community. The authors also incorporated data provided by the YKHC where possible. For dog bite information, all the search platforms were used with primary search term being “dog bite injuries.” Government websites were searched to find state and federal information or legislative information on these topics.

### 2.3 Costs of public health impacts data

Information on the costs associated with rabies exposure, impact of dog bites and dog overpopulation, and costs of implementing a veterinary professional were gathered from literature, governmental websites, HOP operations data, and authors’ personal experiences. Specifically, the World Health Organization (WHO) and Center for Disease Control and Prevention (CDC) websites were searched as well as using the search engine Google to find additional sources was searched for costs associated with rabies exposure using search terms “rabies” and “exposure,” “cost,” and “expense.”

The impacts of dog bites and dog overpopulation data and information were gathered through Google and Google Scholar searches, the NIH website, and online governmental postings of senate bills, press releases, and hearing minutes. Finally, wage information and data were gathered by search the Bureau of Labor Statistics website, with federal salary data specifically from the Office of Personnel Management as well as searching for job openings and associated pay ranges with the USDA. The AVMA Career Center and the AVMA Veterinary salary data tool were also used to gather wage data.

### 2.4 Culminating the findings

The authors worked to incorporate HOP’s experiential data, the completed meta-analysis, and public health impact information a comprehensive summary. Using this summary the authors collaboratively pinpointed and assessed gaps and potential solutions.

## 3 Results

This section examines three distinct but interrelated public health concerns in the YK Delta: (1) exposure to the rabies virus, (2) dog bites to humans, and (3) dog overpopulation. The data collected reveals what public health impacts may occur due to a lack of veterinary medical care, as well as points toward what the costs of implementing a veterinary professional might be. Using a One Health approach, integrative contributions to public health can favor the formulation of policies and the development of strategies (27).

### 3.1 Rabies in Alaska

**Figure 2:**
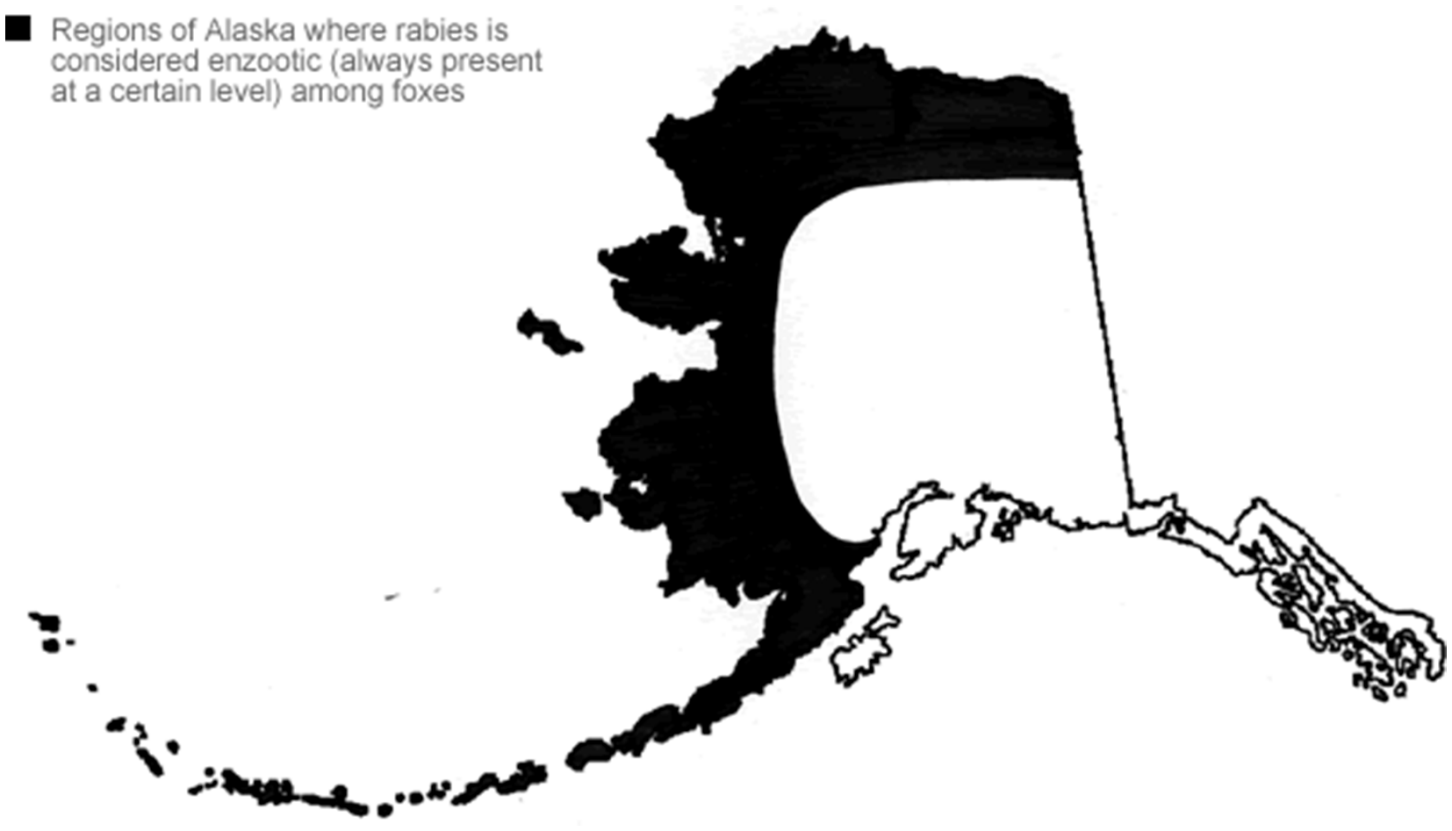
Enzootic regions of AK.

Rabies is enzootic, or always present at a low level, in parts of Alaska as shown in Figure 2 (28). Arctic and red foxes living along northern and western coastal Alaska, including the YK Delta, are reservoirs for rabies (29). Occasionally rabies outbreaks occur in the fox population in the state, referred to as epizootics (30).

Sending suspected cases to a lab is critical for confirmation as rabies can only be diagnosed after death by detecting the virus in the brain. In Alaska, the Alaska State Virology Laboratory (ASVL) in Fairbanks is the only lab that conducts rabies testing. In fact, no Tribal community in the U.S. has a rabies laboratory (10). Thus, they must rely on state or federal laboratories for all rabies testing. All samples from Tribal communities in the YK Delta must be shipped via air to the lab for testing. In certain cases, testing may also be performed by sending samples to the United States Centers for Disease Control and Prevention (CDC) in Atlanta, Georgia (19).

Once an animal is confirmed to have tested positive for rabies, the Alaska Section of Epidemiology (SOE) works with local animal, human, and environmental health partners to identify exposed animals and humans and ensure they receive appropriate follow-up communication and care (31). If a person is bitten or has contact with an animal suspected of having rabies and public safety community officers or healthcare providers are notified, an investigation is conducted to identify the dog, the people and other animals the dog may have encountered. Depending on the type of exposure humans have to the rabies suspect dog, rabies post-exposure prophylaxis (PEP) may be administered. This human rabies treatment consists of a series of shots administered over a defined period. Rabies PEP is indicated for anyone exposed to rabies and should be initiated “as soon as possible” after the exposure (19).

The CDC has conducted several surveillance evaluations to characterize rabies risks in Tribal Lands in the contiguous US and found that rabies testing and reporting rates are up to 15-times lower in several high-risk Tribal communities in the southwestern U.S. when compared to adjacent non-Tribal communities (10).

#### 3.1.1 Rabies prevention

In Alaska, the adopted methods for preventing human rabies are (1) vaccination of dogs and other domestic animals against rabies in rural areas, (2) public education of rabies risk in the wildlife population (e.g., foxes), and (3) prompt administration of rabies post-exposure prophylaxis to persons potentially exposed to the rabies virus (29).

The scope of rabies in wildlife reservoirs in Alaska is currently unknown due to the vast land mass and high wildlife populations in the state (12).

Reducing the occurrence of rabies in Alaskan fox populations is not yet feasible. While oral rabies vaccine baits are approved and available for coyotes and racoons, the oral vaccine baits do not work if they are frozen, thus rendering them ineffective much of the time in Alaska.

Although Alaska State Regulation 7, AAC 27.022, requires all dogs, cats, and ferrets to be vaccinated against rabies (32), this has yet to be achieved in the YK Delta. During 2019–2024, HOP conducted community surveys to collect data on animal and human health, including on reported rabies vaccination rates in dogs. HOP found owner consent to the administration of rabies vaccines to dogs to be high. However, reported dog vaccination rates from owners are far from optimal. In 25 communities visited by HOP, dog owners report only 62% of their dogs had been vaccinated for rabies; when data from Bethel, the largest community with multiple methods to obtain a rabies vaccine, is excluded, only 56% of dogs are reported to have been vaccinated. These statistics come from self-reports by owners and do not assess confirmed or documented vaccination status throughout the 5 years of HOP clinics. Rather, this number is likely a closer representation of dogs that have had a rabies vaccine at one point in their life as opposed to having an up-to-date rabies vaccination. It is expected that the other 33 YK Delta villages not visited by HOP would have even lower vaccination rates as they had even less exposure to veterinary care.

Low rabies vaccination rates in the YK Delta are consistent with the Northwest Territories of Canada where rural community life and geographic challenges are similar. In outreach programs used in Canadian communities, it was found that only 37% of the treated dogs had previously received the rabies vaccine (33).

Due to the lack of consistent access to veterinary medical resources in the YK Delta, rabies vaccination of domestic animals typically occurs one of the following ways: Lay-vaccination program (non-veterinarians), YKHC Office of Environmental Health & Engineering (OEHE) Field Officers, Non-profit veterinary groups conducting community outreach or travel to Bethel with pet to visit veterinary clinic. These are further explained in Table 1.

**Table 1:** Rabies vaccination options for pet owners in the YK Delta, Alaska.

Vaccination option	Comments: availability/frequency of service
Lay-vaccination program (non-veterinarians)	Availability of community volunteers for program. Requires training in vaccine administration and documentation.
YKHC Office of Environmental Health & Engineering (OEHE) Field Officers	Officers visit communities to perform essential duties including, but not limited to, maintaining essential infrastructure, supporting the operation of water and sewer systems in remote communities, injury prevention and educational training. Rabies vaccines administered as time allows.
Non-profit veterinary groups conducting community outreach	Visits are not routine or consistently scheduled. Visit location and timing depends on availability of outreach group.
Travel to Bethel with pet to visit veterinary clinic	Most commonly involves a flight to Bethel. Bethel’s veterinary clinic is typically open 5 days per month.

A less popular but still considered alternative to rabies prevention is culling domestic dogs. However, culling free-roaming dogs or physically removing them from communities has not been found to be effective in controlling rabies (31). It also does not address the underlying transmission of the virus of wild animals to unvaccinated dogs. In addition, dog culling or removal may be unacceptable to local communities. Programs formed with community needs at the center, such as a community’s desire to live with dogs, are found to have more local support and are expected to have better long-term outcomes (32). In contrast to culling, vaccination programs against rabies in dogs have proven efficacy and feasibility across a wide range of settings and raise far fewer ethical or welfare issues (33).

In a dog census completed by HOP, personnel went door-to-door to ask residents about the number of dogs they owned. As shown in Table 2, a total of 140 dogs were counted in Quinhagak, human population 750; 102 dogs in Napakiak, human population 344; 166 dogs in Akiak, human population 494. The percentage of dogs per person in these three communities ranged from 18.6 to 33.6% of dogs per person.

**Table 2:** 2020 community census of owned dogs from three YK Delta villages.

Village	Dog population	Human population	Dog/human %
Quinhagak	140	750	18.6%
Napakiak	102	344	29.7%
Akiak	166	494	33.6%

These numbers are nearly identical to the dog/human population overall in the United States. In 2017, there were approximately 89.7 million dogs owned in the United States (34), and in 2017 the US general population was an estimated 326 million (35). This is a dog/human percentage of 27.6%, comparable to the dog/human percentage of YK Delta villages.

Dog owners in the YK Delta region generally support rabies vaccinations in their dogs. As observed by the HOP team, nearly all owners consent for their dogs to be vaccinated against rabies. This was true for both of the communities that had confirmed animal rabies cases, Quinhagak and Napakiak, as well as the community that was part of the census via a routine HOP community visit, Akiak. HOP visited Quinhagak in April 2020 and Napakiak in May 2020 at the request of the community members and local officials following confirmed rabid wildlife entering the community (coyote and fox) (36). Typically, YKHC OEHE personnel respond to these events. However, at that time staff of those were overburdened due to response measures put in place early in the COVID-19 pandemic.

The large-scale vaccination of humans to prevent or decrease the impacts of infection, to other viruses (i.e., influenza), is an alternative. In fact, pre-exposure rabies vaccination (PrEP) is recommended for persons in high-risk groups, such as veterinarians and their staff, animal handlers, rabies researchers, and certain laboratory workers. However, it is not used in the general public due to low rabies rates in most of the U. S (10). Additionally, PrEP does not replace the need for PEP. Any person exposed to a suspected rabid animal, even if they have received PrEP, needs to still seek post-exposure care (10).

#### 3.1.2 Costs associated with rabies exposure

Suspected rabies exposures exert economic burdens on local municipalities, county governments, and healthcare systems in rabies endemic areas (37). Not all losses from zoonotic diseases such as rabies are preventable. In some cases, effective vaccines do not exist for all the main vectors and, in the case of highly contagious zoonoses, large outbreaks can happen rapidly and involve thousands of people simultaneously. In addition, for those zoonotic diseases that have high morbidity implications, illness can persist for an extended period, causing economic impacts for many years. Rabies, however, is unique, in that a vaccine does exist for dogs, the most common route of human exposure (38).

There are several types of expenses associated with exposure to rabies. These range from direct treatment costs borne by the patient or the health system to indirect costs to the individual, their family, and the community. In the United States in 2017, the reported mean total cost (considering direct and indirect costs) of a suspected human rabies exposure was $3,688 (range $721–$9,197) (39). Adjusting for inflation in 2024, those estimated costs are $4,683.93 (range $1,225–$16,016) (40). Yet some patients report costs of over $25,000 (41). *Alaska’s* per capita expenditures on hospital care are approximately 50% higher than the national average, and about 80% higher for physician and clinical services (42).

In an in-house report shared with HOP, YKHC OEHE reported that over the past 13 years, 1,522 rabies investigations were conducted following reports of dog bites. Of these a total of 182, or 14.8%, of the cases were recommended for rabies post-exposure prophylaxis (Table 3). The rabies post exposure prophylaxis (PEP) protocol for immune competent and previously unvaccinated persons, consists of 4 doses of rabies vaccine and 1 dose of immune globulin administered over a 14-day period (10). Critically, PEP only prevents the development of rabies in humans and does nothing to address the source of exposure. Administering the treatment is also time-sensitive and time-consuming for professionals, patients, and families.

**Table 3:** YKHC rabies investigations and PEP advised 2012–2024.

Year	Number of investigations	Number of PEP’s advised	Percentage of PEP’s advised
2012	81	5	6.2%
2013	76	6	7.9%
2014	93	12	12.9%
2015	103	11	10.7%
2016	106	12	11.3%
2017	123	8	6.5%
2018	144	25	17.4%
2019	202	26	12.9%
2020	92	17	18.9%
2021	141	24	17.0%
2022	145	33	12.4%
2023	148	25	16.9%
2024 as of 12/9/2024	153	36	23.5%
TOTAL 2012–2024	1,522	182	14.8%

Indirect costs can account for a significant portion of overall costs of rabies exposure. In fact, Shwiff et al. (39) found that about one third of the total cost for suspected human rabies exposure was attributed to indirect costs (e.g., time, lost wages, transportation, and day-care fees), most of which are not reimbursable to the patient. An example of multiple indirect costs comes from a case reported to HOP from the YK Delta. A family of five living in a remote village needed PEP treatment following exposure to a rabid dog. The family was flown to and lodged in Bethel, AK throughout their two-week course of treatment. Further, in small communities like these, indirect costs can have a ripple effect. In this case, one of the family members was a teacher in the village school where there is often only one teacher and grades K-12 may be combined. With no substitute teacher available while the teacher was receiving treatment in Bethel, the school had to close and parents had to attend to children instead of working, ultimately affecting the entire community.

Another potential indirect cost of rabies exposure is mental health expenses. The psychological effects of rabies exposure can result in counseling or treatment for anxiety after being exposed to a potentially rabid animal and needing to undergo treatment for a potentially fatal disease. This may occur as patients, families and providers may experience distress during post-exposure risk assessments and PEP administration (43). Additionally, while in the lower-48, animals may be quarantined to see if they develop signs of rabies, however, in the YK Delta, facilities and personnel to quarantine such dogs are not available and the risk of rabies is high, rendering this option unworkable. Therefore, unvaccinated dogs suspected of rabies exposure, as well as dogs suspected of being exposed to a rabid animal and whose vaccination status is uncertain, are culled to test for rabies or to prevent further spread of the disease, impacting the humans they are associated with. The responsibility for culling these dogs falls on the Village Safety Public Officer, who may know the dog and/or the family that owns it, adding to the potential distress and negative mental health outcomes for additional individuals and the entire community.

The costs of human rabies exposures discussed above cover many, though not all, of the potential costs associated with the disease. To date, a full assessment of all costs remains absent from the literature (39). Such a study would need to include the psychological costs to individuals who are not suspected of being exposed to rabies but who may have to deal with possibly rabid animals (i.e., Village Safety Public Officers culling animals or YKHC OEHE employees completing rabies investigations), the cost of animal testing including those costs associated with packaging and shipping samples to the laboratory, as well as the indirect costs related to the need to educate the public on the risks of rabies exposure as well as public health workers.

#### 3.1.3 Costs of rabies prevention

The literature review, based on several papers focused on a variety of global regions where the cost of rabies control had been estimated for a particular community, revealed that rabies is an economically significant zoonosis. In many cases, the decrease with intent of elimination of rabies exposure through vaccination of canines proves more cost-effective over time than the continual administration of PEP, animal testing and animal vaccination (37). Annual canine rabies vaccination campaigns have shown to be valuable and reduce the health burden of rabies. Studies that illustrate this include work done in Serengeti and Ngorongoro, communities in sub-Saharan Africa, which found annual canine rabies vaccination to be broadly cost-effective in both regions. It was found that even high-coverage annual vaccination campaigns in Serengeti are cost-saving compared with PEP alone within the first year (44). Another study found that after 15 years a canine robust rabies exposure program consisting of a combination of PEP and canine vaccination becomes more cost effective than PEP alone (45).

Further, it has been found that the cost of annual campaigns could be reduced by about 15% over a decade by eliminating repeat vaccinations. Once a pet has completed the initial vaccination series (one after 12 weeks of age, and one the following year), dogs may be unnecessarily brought back each subsequent year for revaccination (46).

Another mechanism for making rabies control more cost effective comes from the WHO which reports efforts should be made to fully incorporate rabies control activities in all levels of public health services, aligning them with other public health programs such as those for tuberculosis. In this manner, synergies between programs improve logistical use of human, material, and financial resources (18).

HOP’s experiential data demonstrates several of these literature findings. Vaccinations were provided by local partners YHKC OEHE while HOP provided the personnel to administer the vaccinations, record client and patient information, and distribute rabies tags and vaccination certificates. Vaccination data was shared back with YKHC OEHE. This partnership allowed increased efficiency of a systematic vaccination campaign in each of the visited communities.

### 3.2 Impact of dog bites

Notably, Alaska Indigenous children are hospitalized due to dog bites more than anyone else in the Indian Health Service system, which serves tribal communities across the U.S. (47). The YK Delta has seven to nine times the annual national average of per capita reported dog bites in all children (48). A 2014 study ranked dog bites as a top 10 priority for injury prevention among Indigenous children in Canada (49).

The HOP can provide up-to-date insight to the relevant reports on dog bites in the YK Delta. In a paper, *Community Perspectives on Dogs, Health Risks, and Veterinary Care Impacts in Rural* Alaska (50), HOP surveyed YK Delta residents from communities visited about dog bites in their communities. As shown in Table 4, among the 375 survey respondents, 39.2% felt dog bites and attacks were a problem in their communities, and Table 5 shows nearly 42% of respondents reported being bitten by a dog. However, not all dog bites are reported to health care workers (51).

**Table 4:** Survey: are dog bites a problem?

Q: Are dog bites or attacks a problem in your community?		
Yes	147	39.2%
No	81	21.6%
I do not know	147	39.2%
*N*	375	

**Table 5:** Survey: self-reported dog bites.

Q: Have you ever been bitten by a dog?		
Yes	156	41.8%
No	204	54.7%
I do not know	13	3.5%
*N*	373	

Not only does dog bite prevention generally increase positive public health outcomes in a community, it is also key to rabies prevention. Community-based educational interventions in conjunction with veterinary services (dog vaccinations and sterilization surgeries) have been shown to help decrease dog bites (52). HOP also implemented these practices, visiting the village K-12 school and offering education sessions on veterinary medicine and dog bite prevention.

### 3.3 Population control

In the same paper on Community Perspectives, analysis reveals that most community members believe stray/unwanted dogs are a problem (71.2%, Table 6), and report counting ten or fewer loose dogs per day (approximately 82%), with some reports of more than 25 (approximately 5% of respondents). Most community members report that dogs do not typically have identification (47.7%), about a quarter reporting that they do have identification (28.2%), and 24.1% are unsure if they have identification.

**Table 6:** Survey: are strays or unwanted dogs a problem in your community?

Q: Are strays or unwanted dogs a problem in your community?		
Yes	267	71.2%
No	25	6.7%
I do not know	83	22.1%
*N*	375	

The literature indicates that some of the most typical approaches to managing dog populations include sheltering, culling, and fertility control (26). Creating sheltering services for free-roaming dogs is common in the United States, and 59.8% of these shelters have a veterinarian on staff (53). The sheltering strategy uses removal of dogs from the area, sometimes accompanied by sterilization surgeries, to reduce the free-roaming dog population size.

Literature on culling, the episodic removal and killing of animals for population reduction, has found that although it resulted in an initial decrease in dog population size (e.g., 5 years), fertility control was more effective at reducing the population size over longer periods (e.g., 20 years) (54).

Fertility control, achieved through surgical or chemical sterilization or contraception, has had the greatest reported effect on dog population control. The strategy of using spay/neuter (surgical sterilization) is to decrease dog population size by preventing births, therefore allowing a reduction of numbers as natural deaths occur. Spay/neuter sterilization specifically was found to be the most effective single method of addressing overpopulation, particularly over long-time horizons (55). In observational studies, the impacts of fertility control varied from decreases in population size of 12% in 1.5 years to decreases in size of 40% over 12 years (55). The arguments for population control over culling is supported by the WHO which has published guidelines discouraging the use of culling and instead recommends alternative methods to control population numbers (e.g., registration and identification, vaccination, public education, and sterilization) (18). The HOP used the strategy of population control in the YK Delta by performing 1,203 spay/neuter surgeries between June 2019 and August 2024 and the results from a HOP community survey found 73% of respondents who responded to a question on what has changed in their communities since HOP visited was about population control.

One respondent in the community survey mentioned an increase in safety. In fact, the issue of population control as it overlaps with public health is visible in both HOP experiential data and the literature. Communities have reported to HOP issues with dog overpopulation include dogs spreading garbage, fecal contamination of soil and water, and loose dogs getting into stored subsistence food (fish, moose, caribou) that families depend on to survive in rural Alaska. Further, some literature finds that fertility control can reduce public health risks. The Association for Animal Welfare Advancement found that there is an improvement of public attitude towards the presence of free-roaming dogs after fertility control (53). Smith et al. found that fertility control of 65% of females over an unspecified length of management was associated with a significant reduction in human bite cases of five bites per month (26).

### 3.4 Costs of implementing a public health veterinary program

As of the date of this publication and to the best knowledge of the authors, there has not yet been a veterinary professional implemented into the human medicine system of health care in the U.S. Therefore, there is a dearth of literature on the subject. This section describes many of the main components of cost that would come into play in the implementation of such a position and program.

While the following costs listed below are not comprehensive, they serve as a starting place when looking at implementing a public health veterinarian program, including the veterinarian’s compensation, a veterinarian technician’s compensation, travel, equipment and supplies.

#### 3.4.1 Veterinarian compensation

According to the United States Bureau of Labor Statistics, the annual mean wage for veterinarians in Alaska is $129,820–$183,860 (56). The *American Veterinary Medical Association Veterinary Salary Estimator* uses AVMA economic data to allow users to apply different variables such as years of experience, practice type, and completion of a residency program to generate salary estimates (57).

The AVMA Veterinary Salary Estimator was used to explore salary ranges for a veterinarian practicing veterinary public health in Alaska with a tribal or other governmental organization which can be seen in Table 7. The table shows four configurations of salary using variations in years of experience, either 5 or 10 years, and completion of a residency, yes or no (a master’s in public health degree (MPH) could be equivalent to a residency for a public health veterinarian). The tool found a similar pay range to that of the Bureau of Statistics, though the lowest end was much lower ($75,325) and it is unclear if the AVMA Estimator uses a mean for its estimations.

**Table 7:** AMVA veterinary salary estimator results.

AMVA veterinary salary estimator results			
Years of experience	Practice type	Residency Y/N	Salary range
5 yrs	Federal/state/local	N	$75,325–$108,573
5 yrs	Federal/state/local	Y	$102,516–$147,878
10 yrs	Federal/state/local	N	$83,551–$124,050
10 yrs	Federal/state/local	Y	$128,955–$181,955

Before a veterinarian can begin to provide services in a program such as this, they must secure individual professional liability insurance policy, receive their state veterinary licensure, and obtain a DEA license to purchase the necessary medications for surgery. These costs amounted to approximately 1,300 per year in 2024 for a veterinarian in Alaska. Specifically, the annual professional liability insurance policy was $434, an Alaska State Veterinary License fee carries an initial fee of $800 and renewed every 2 years at $600; and a DEA license of $888 for a three-year term. In addition to salary, veterinarian compensation may include benefits such as health, dental and vision insurance, retirement contribution and paid leave.

#### 3.4.2 Veterinary assistant or technician

Similar to human health use of nurses and health technicians a veterinarian in the U. S. needs a veterinary support staff, either trained (assistant) or licensed (technician) to perform their professional services (examinations, treatments, surgery). According to the Bureau of Labor Statistics in 2024, the annual mean wage of a veterinary technician in Alaska is $45,510 (56). Veterinarian technician compensation may also include benefits such as health, dental and vision insurance, retirement contribution and paid leave.

#### 3.4.3 Travel

A public health veterinarian often needs to travel, particularly to rural locations. The cost of travel to these rural locations needs to be considered in the overall cost of the public health veterinary program In the lower-48, airplanes and vehicles are accessible and commonly used. In other locations such as the YK Delta, travel occurs in small aircraft or boat. March 2025 published rates for roundtrip travel per person to communities located in different areas of the YK Delta on a local regional carrier indicate that, when flying as a ticketed passenger (rather than chartering a plane), A single round-trip ticket on average costs $600 (Table 8) (58). Unanticipated trips in response for emergency services, such as rabies outbreaks, require extra funds in addition to planned trips.

**Table 8:** Commercial roundtrip airfare from Bethel, Alaska to selected YK Delta villages (March 2025).

Destination	Roundtrip cost
Emmonak	$730
Hooper Bay	$650
St. Mary’s	$560
Toksook Bay	$460

#### 3.4.4 Equipment and supplies

Supplies and equipment will be highly variable depending on the scope of a program. HOP experiential data suggests a total of $25,000 in supplies annually to administer a similar program. Between 2019–2024, this encompassed 108 clinics across 25 communities and services for 4,884 animals. Services included distemper/parvovirus vaccination, antiparasitic treatments, and 1,203 spay/neuter surgeries. However, this does not include rabies vaccinations which were provided by YKHC, estimated at $24,000, and an initial supply donation in 2019 estimated at $10,000. This donation included some critical items such as an anesthesia machine, oxygen concentrator, surgical instruments and safe for controlled medication needed for surgery. Further, other consumable supplies were donated such as suture, surgical drapes, etc., which would lead to an increase in estimated cost annually for a similar program.

However, the cost may decrease over time, particularly for rabies prevention. It is clear from other studies, that the number of rabies vaccinations administered, and the frequency of vaccination efforts decreases as the number of dogs in a community are vaccinated. Once vaccination coverage reaches a certain level, the number of dogs receiving an initial rabies vaccine would drop significantly and the emphasis will shift to vaccinating puppies and providing annual vaccines (59).

## 4 Discussion

The general US population appears to own as many dogs as community members in the YK Delta. Yet the general US population may be able to drive to veterinary clinics, find a community animal shelter, or explore local options such as animal welfare organizations. These are lacking in the YK Delta and other similarly situated underserved areas.

This research suggests that the high costs of rabies exposure, inefficient rabies prevention, dog bites, and population control are worthy of an investigation of solutions. Importantly, the YK Delta and much of Alaska is distinct from much of the lower-48 and requires a distinct solution. Rabies is endemic, and there is a reality that people are routinely exposed to the virus when their dogs encounter potentially rabid wildlife. Other public health concerns, including dog bites and overpopulation, also demonstrate the need for a sustainable solution. Due to the costs and barriers outlined above, the authors recommend incorporating a permanent community-based public-health veterinarian or veterinary program into the human healthcare system in the YK Delta.

### 4.1 Implementing a public health veterinary program

This public-health veterinarian position and program is a long-term sustainable solution to the public health issues encountered by dogs in the YK Delta. In Alaska, this would occur through the Indian Health Services which currently provides healthcare to these villages. While some organizations are currently doing this work, such as HOP, they are largely non-profit or grant funded, with funding inconsistencies, thus leading to inconsistencies in delivery of services.

Currently, the costs of rabies PEP, dog bite investigations, dog bite injuries, all of which are tied to population control, spread the financial burden across individuals, communities, insurance companies and health organizations. The public-health veterinary program approach to providing veterinary care would provide consistent rabies prevention, along with other disease vaccinations, ultimately lowering the rate of human exposure; and they would coordinate regular spay and neuter services for population control which would lead to lower rates of dog bites and rabies exposure, as well.

To be sure, public health veterinarians provide services in addition to those related to rabies exposure, dog bite prevention, and population control. They are a resource of information and knowledge on other zoonotic diseases and food safety; they can be deployed to specific incidents, including disaster response and disease outbreaks; and they coordinate with other officials and agencies on public health issues, such as avian influenza.

A large part of the total cost of implementation of this plan will be the cost of salaries and benefits for a veterinarian and veterinary assistant or technician. The cost of travel, including airfare and lodging as well as equipment and supplies will vary depending on the needs of the communities served and the number of visits annually. As of 2025, based on HOP experiential data, the authors recommend a starting point of a minimum of $25,000 annually for supplies.

Travel to each community should occur on an annual basis. The technical skills of the support staff member play a critical role for clinics, but they may also play a key role in overseeing the logistics of travel and necessary record keeping including a standardized process for rabies vaccinations. Travel expenses typically include lodging, flights or fuel and meals. One benefit of partnership in an existent hub and model medical system is that there is potential to use the same lodging support system as for other traveling YKHC healthcare providers.

A consistent, full-time veterinarian and support staff providing care and tracking annual community visits can achieve significant savings for the program by implementing in a number of systems. These systems include improved record-keeping practices, a unified identification system for owned dogs, and/or discouraging serial canine vaccination, as demonstrated in the work completed in sub-Saharan Africa.

### 4.2 Benefits of implementing a public health veterinary program

One reason a public health veterinarian may not already be embedded in the human healthcare system is that while it is easy to look at the cost of veterinary services, no work to date has identified the comprehensive costs of a lack of public health veterinary services. The authors have made an initial, broad stroke attempt at this in the section above. Here, the benefits of implementing a permanent community-based public health veterinarian in the region are presented. This implements the One Health approach as a means of controlling and preventing zoonotic diseases, considering the results found in this study.

First, a permanent community-based public-health veterinarian or veterinary program in the region would allow for the coordination of existing prevention initiatives (e.g., lay vaccinators, OEHE staff, non-profit veterinary groups), the development of a plan to identify where and when villages need visits to ensure access to domestic animal rabies vaccine for all communities.

Like the lack of access to veterinary medical services in similar Canadian communities, lack of accessibility is a key factor for the YK Delta’s low vaccination rates. The benefits of annual canine rabies vaccination campaigns seen in the literature can be applied through this position in the YK Delta which shares geographic characteristics to those communities such as being in remote, rural regions with lower resources.

Further, we know addressing the enzootic rabies issue through effort in human health, such as large-scale vaccination of humans, is unrealistic for reducing the health burdens. Any person exposed to a suspected rabid animal, even if they have received PrEP, needs still seek post-exposure care. The use of this method would only increase the cost associated with the disease.

Not only do annual canine rabies vaccination campaigns reduce the health burden of rabies, but the repeated annual canine vaccination against rabies, particularly with record documentation and signaling on dogs, is also cost saving. The permanency of this position allows for consistency and up-to-date record keeping.

Second, a public health veterinarian is optimally situated to provide community-based educational interventions in conjunction with the above veterinary services (dog vaccinations and sterilization surgeries) which are shown to help decrease dog bites. A decrease in dog bites comes with a strong potential for a decrease in rabies exposure.

The prevalence of dog bites as reported by HOP is consistent with the literature on indigenous communities in Canada. It is likely that the numbers reported by HOP are underestimated as the reporting structure only captures incidents severe enough to warrant travel to a clinic for treatment. This underestimation may be obscuring the full public health burden.

The risk of rabies transmission is very real as dog bite incidents are high with suboptimal canine vaccination rates in a region endemic for rabies. The response to dog bites and rabies remains reactive in this region, however, primarily deploying post-bite risk assessment and PEP treatment. This is concerning as not all dog bites are reported to health care workers.

Third, regular spay/neuter campaigns led by a public health veterinarian can greatly reduce the free-roaming population of dogs. A permanent veterinary position allows for consistency in sterilization clinics and will avoid gaps in coverage, which can lead to population booms.

Understanding the nuanced dynamics of dog population in the YK Delta is important to understanding the problem of overpopulation. The presence of free-roaming dogs can be considered an issue in terms of public health (e.g., transmission of rabies and other zoonotic pathogens), impacts to wildlife and the environment, and the animals’ own welfare state. Both owned and unowned free-roaming dogs are particularly important to population control as the lack of physical restriction allows them to freely roam, mate, and reproduce. While sheltering is a common approach, it is not realistic in the YK Delta due to the small community size (average populations of 100–1,000), an overabundance of dogs, and a lack of resources.

Finally, an increase in vaccination rates will lead directly to a decrease in rabies exposure outcomes including the total monetary burden on both individuals and the human healthcare system and distress in communities who cherish their dogs. Further, the program can include a system for increased testing efficiency, improving the exposure processes overall.

Threshold vaccination coverage of dogs benefits the community by greatly reducing the risk of rabies exposure from their dogs, and saves individuals, the community and the health care system the direct and indirect costs associated with treating a dog bite and potential rabies exposure.

Mental health impacts and expenses due to rabies exposure (e.g., counseling or treatment for anxiety) is yet another example that supports why systematic vaccination of dogs against rabies is preferable to systematic reliance on PEP to prevent rabies in people. The experience of being exposed to a potentially fatal disease is distressing. Additionally, unvaccinated dogs suspected of rabies exposure are culled to test for rabies or to prevent further spread of the disease, impacting the humans they are associated with.

The authors further believe that the CDC’s assessment which found rabies testing and reporting rates are up to 15-times lower in several high-risk U.S. Tribal communities can be extrapolated to the Tribal communities in Alaska which share many characteristics with those in other parts of the U.S. (e.g., rural, remote, low resourced, etc.). A public health veterinary program could focus on implementing a system to facilitate reporting processes and increase accessibility to testing.

The cost of treating rabies exposure in the YK Delta or a similar remote location is greater than when a similar treatment is given in other parts of the lower-48 states. Administering the treatment is also time-sensitive and time-consuming for professionals, patients, and families. For residents of remote villages in the YK Delta requiring treatment, this likely means traveling to Bethel and staying there through the completion of the two-week course of treatment. This was demonstrated in the HOP data with the story of a family of five in a remote village who needed to fly to and lodge in Bethel for 2 weeks. This left the village without an operational school and ultimately, the entire community was affected as parents then had to attend to children instead of working their jobs, on top of the school children losing 2 weeks of educational access. Table 9 shows costs, when available, of these public health issues, since so much of this data is not available, further research is needed.

**Table 9:** Initial public health related veterinary costs.

Costs associated with rabies exposure	Per Person Rabies cost in USD when available	Costs associated with dog bites	Dog bite cost in USD when available	Costs associated with dog overpopulation	Dog overpopulation cost in USD when available	Annual Costs associated with a public health veterinarian	Public health veterinarian cost in USD when available
Bite investigation	N/A	Bite investigation	N/A	Potential for increase in dog bite injuries	N/A	Veterinarians’ salary	$156,840
Culling and testing of animal	N/A	Average individual dog bite insurance claim	$58,545	Potential for other zoonotic disease spread	N/A	Veterinarians’ benefits	N/A
Transport of person from village for treatment	~ $600			Community health impacts (dogs spreading feces, garbage)	N/A	Veterinarians licensing and dues	$10,300
Time away from work, family and community	N/A			Dogs getting into stored food essential to village life	N/A	Support staff salary	$45,510
Estimated individual Rabies PEP medicine	~$7,755			Emotional well-being tolls in a community (dog culling)	N/A	Support staff benefits	N/A
						Travel to each community	~ $600 per person per trip
						Clinic supplies	$25,000

While there are multiple ways to come to an estimate of cost, the authors took an approach based on the framework of HOP. HOP was specifically designed to bring public health veterinary care to the YK Delta, therefore this paper is focused on the region HOP served, the stakeholders it worked with, and the services it provided. The authors, then, use data provided by the YKHC where possible and consider the unique characteristics of the YK Delta region to paint a picture of what the healthcare costs may look like for those living in the YK Delta.

Starting with the 2017 United States reported mean total cost of a suspected human rabies exposure of $3,688, adjusted for inflation to 2024, is about $4,700 for the lower-48 states. Since *Alaska’s* per capita expenditures on hospital care are approximately 50% higher than the national average, and about 80% higher for physician and clinical services, this paper uses a midrange estimate of $7,755 per person for the cost of the PEP antidote administration alone ($4,700 and a 65% cost increase in Alaska).

On a larger scale, extrapolating the midrange estimate for rabies PEP treatment above, in combination with the 182 rabies investigations cases which were recommended for rabies post-exposure prophylaxis over 13 years by the YKHC (Table 3), an estimated cost of $279,180 was incurred to the healthcare system in the YK Delta in 2024 for rabies PEP antidote administration alone. The overall costs are surely increased in the YK Delta once you consider the other aspects distinct to the region noted above: travel and lodging costs, time away from work and family, and time lost from subsistence activities that provide food.

PEP is only one aspect of the overall costs of rabies exposure, as discussed above. Compare this figure of $279,180, which encompasses only PEP within the YK Delta region, to the estimated costs of running a public health veterinary program as laid out in Table 9. Assuming there are two people (veterinarian and technician or assistant) who complete 12 trips per year, the annual cost of the program would be approximately $252,050. This total will fluctuate depending on the actual number of people traveling and the number of trips completed. However, it is clearly more cost-effective to have a pro-active public health veterinary program embedded into the larger health care systems than to continue to be reactive to the issue, leaving the individuals, families, communities, organizations and society with the cost burden. The program could serve more communities than HOP had the capacity for and reduce overall costs.

### 4.3 Steps toward implementation

The authors strongly suggest that future work on this topic addresses how to implement such a position and its potential barriers. Policy changes will likely be necessary to fully incorporate a veterinarian into the human health system to function at the level envisioned in this paper, and the federal landscape at the time of this writing may not be conducive to such changes.

However, there are some tangible steps the authors believe could be considered in moving forward with such a program. First, discussions with regional healthcare corporations and governments should be conducted regarding developing and adding a position for a public health veterinarian within their organization(s). Second, advocates can be identified who can work with Congress and/or Indian Health Services (IHS) to create and fund a public health veterinarian position with the IHS. This would make having a public health veterinarian an option for all tribal nations served by IHS. Finally, leveraging the fact that there are currently veterinarians working in public health positions in the federal government and in many states. Many states, including Alaska, have added a Public Health Veterinarian position in the state government to integrate veterinary health and human health issues and solutions. This provides a pathway for other governmental organizations interested in creating such a role (60).

## 5 Conclusion

In most cultures, including the Alaskan Native communities in the YK Delta, dogs not only provide companionship, but they are also woven into the social fabric of everyday life. Yet the lack of access to veterinary care is an ongoing public health crisis that is severely impacting the quality of life for people and animals. HOP demonstrates the need for public health veterinary services and a model of what sustained focused regional veterinary care can look like.

A public health veterinarian providing integrated veterinary services are critical. To date, without a permanent program or position such as this, access to veterinary care in rural Alaska, particularly Alaska Native communities, has been scattershot. Further, there is a want and a need for a public health veterinarian to be incorporated into the existing human healthcare system by the residents of this region. In October 2022 the Alaska Federation of Natives created Resolution 22–9 that states, “[t]he lack of veterinary care is an unmet public health crisis in Alaska Native Communities and [we] call to have veterinary service be included under Indian Health Service purview” (61). It is clear the communities in this region support inclusion of veterinary healthcare in their public health services.

While the lack of veterinary care impacts public health, providing it is a means by which public health is improved. Preventative veterinary medicine can be considered a low cost compared to the high cost of disease.

HOP demonstrated the effectiveness of providing consistent veterinary preventative care and how that can increase the number of vaccinated dogs, thereby reducing rabies exposure risk to humans. In addition, education and population control provided by HOP can decrease the number of dog bites and dog overpopulation.

More consistency over the long term will demonstrate even better results. An added benefit of having a dedicated public health veterinarian to provide preventative veterinary care is the broad training the public health veterinarian has in addressing other public health threats, including other zoonotic diseases, food safety and prevention of food borne disease.

One foreseen challenge will be the integration of public health veterinary services into existing infrastructure and ongoing health programs. Currently, health authorities must balance the investment of scarce resources and veterinary programs are often perceived as low priority. Some medical professionals are currently unaware of the “One Health” approach, and it is important to increase its dissemination. Results demonstrate the tremendous human health benefits when public health veterinary services are available, from reduction in rabies exposure, decrease in dog bites and population control.

### 5.1 Limitations

**Figure 3:**
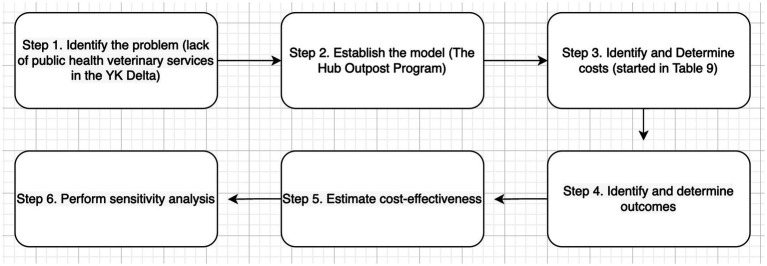
Steps in cost-effectiveness analysis Adapted from Petitti.

This paper begins the work of assessing some of the costs associated with not having a public health veterinarian to respond how dogs impact on public health in the YK Delta of Alaska. However, the authors have not conducted a true economic cost: effectiveness analysis. It is critical to understand the costs of available interventions as a vital consideration in policymaking within healthcare. Such an analysis is recommended to further examine true costs when access to public health veterinary medicine is not available in YK Delta. Using an adopted diagram (62) Figure 3 as a guide, future cost-effective analyses may begin using data from HOP in the first three steps.

Further, there are multiple other methods to estimate or discuss the potential cost of rabies control. These include, but are not limited to, an epidemiological model, stakeholder analysis, and pilot programs. Epidemiological modeling uses disease modeling techniques to simulate rabies transmission and control. This can help estimate the costs of various intervention strategies and their potential effectiveness. A stakeholder analysis can help to identify and analyze different perspectives of stakeholders involved (e.g., governments, healthcare providers, animal control agencies, etc.) to better understand how costs are distributed and what potential funding avenues are available. Pilot programs implement new rabies control programs and methods in selected areas, monitor the costs and outcomes, and use this data to estimate broader potential costs.

While there are 58 communities in the YK Delta, HOP was only able to visit only 25 due to resource restrictions associated with budget, time, and personnel. A public health veterinary program for the entire region would need to ensure a delivery of resources across the region.

## Data Availability

The raw de-identified HOP data supporting the conclusions of this article will be made available, by the authors, upon request without undue reservation. All other data is provided in the references section.
